# Prevalence and Distribution of Endosymbionts in *Bemisia tabaci* Populations from Pakistan: Dominance of *Arsenophonus* in Indigenous Asia II-1 Population

**DOI:** 10.3390/insects17060585

**Published:** 2026-06-03

**Authors:** Mariyam Masood, Zafar Iqbal, Roma Mustafa, Sallah A. Al Hashedi, Adil AlShoaibi, Rob W. Briddon

**Affiliations:** 1 Agricultural Biotechnology Division, National Institute for Biotechnology and Genetic Engineering, Faisalabad 44000, Pakistan; rob.briddon@gmail.com (R.W.B.); 2 Center for Advanced Studies in Agriculture and Food Security, University of Agriculture, Faisalabad 38040, Pakistan; mariyam.masood@uaf.edu.pk (M.M.); 3 Central Laboratories, King Faisal University, Al-Ahsa 31982, Saudi Arabia; sahmad@kfu.edu.sa; 4 Department of Biotechnology, Sardar Bahadur Khan Women’s University of Quetta, Quetta 87300, Baluchistan, Pakistan; roma.mustafa@sbkwu.edu.pk; 5 Department of Physics, College of Science, King Faisal University, Al-Ahsa 31982, Saudi Arabia; adshoaibi@kfu.edu.sa

**Keywords:** *Bemisia tabaci*, endosymbionts, begomovirus, cotton, biotypes, cryptic species

## Abstract

*Bemisia tabaci* is a globally destructive agricultural pest and an efficient vector of begomoviruses, which cause recurrent epidemics across South Asia, including cotton leaf curl disease in Pakistan. Increasing evidence shows that bacterial endosymbionts play a central role in shaping whitefly biology, population dynamics, and vector competence. However, the distribution of these symbionts remains poorly resolved in Pakistan, a region where begomoviruses are persistent and widespread. This study investigated the cryptic species diversity, secondary endosymbiont composition and their infection frequency in *B. tabaci* populations collected from major agricultural regions of Pakistan. A total of 274 adult whiteflies belonging to Asia II-1 (*n* = 199), MEAM-1 (*n* = 67), Asia I (*n* = 7), and Asia II-8 (*n* = 1) were screened using a symbiont-specific PCR assay for six endosymbionts. The primary endosymbiont *Candidatus Portiera aleyrodidarum* was detected in all individuals, whereas five secondary endosymbionts (*Arsenophonus, Cardinium, Hamiltonella, Wolbachia and Rickettsia*) were identified with distinct cryptic species- and region-specific patterns. Notably, *Arsenophonus* was the most prevalent endosymbiont, occurring in 68% of Asia II-1, 100% of Asia I, and 21% of MEAM-1 individuals, with the highest regional prevalence in Punjab (80%) and Khyber Pakhtunkhwa (77%). Logistic regression analyses confirmed significantly higher infection probabilities in indigenous Asia II-1 populations. Network analysis revealed structured co-occurrence patterns, including strong negative associations between *Arsenophonus* and *Hamiltonella*. Phylogenetic analyses revealed close relatedness of Pakistani *Arsenophonus* strains to those reported from neighboring regions, indicating regional community rather than unique local diversification. The dominance of *Arsenophonus* in Pakistani whitefly populations is of particular significance, given its role in protecting begomoviruses within the insect vector and its implication in facilitating virus persistence and transmission. This study, for the first time in Pakistan, provides a comprehensive assessment of endosymbiont–cryptic species associations in Pakistani *B. tabaci* populations and highlights the dominant prevalence of *Arsenophonus* as a potential key player in local virus vector dynamics.

## Simple Summary

Whiteflies are tiny insects that cause major damage to crops by feeding on plants and spreading plant viruses that reduce yield and quality. In Pakistan, these insects are a key reason for recurring disease outbreaks in important crops such as cotton. While much attention has focused on the insects themselves, less is known about the bacteria living inside them and how these microbes influence their ability to spread disease. This study examined whitefly populations collected from major agricultural regions of Pakistan to understand which bacteria they carry and how these are distributed. We found that most whiteflies harbor one or more types of bacteria, with one group, called Arsenophonus, being the most common, especially in the dominant local whitefly type. This bacterium was particularly frequent in regions where plant virus problems are severe. Our results suggest that these internal bacteria may influence how effectively whiteflies transmit plant viruses, although further work is needed to confirm this role. Understanding these hidden microbial partners provides new insight into why plant diseases persist and spread, and may help develop improved, environmentally friendly strategies to manage whiteflies and protect crop production.

## 1. Introduction

The whitefly (*Bemisia tabaci*) is one of the most economically important insect pests distributed worldwide and represents a cryptic species complex consisting of at least 45 morphologically indistinguishable species [1,2,3]. *B. tabaci* is a highly polyphagous insect infesting a wide range of agricultural and horticultural crops. It causes direct damage to the plants by sucking the phloem and indirect damage by secreting the honeydew which promotes the growth of sooty mold and reduces photosynthetic efficiency. Most importantly, *B. tabaci* is a vector of several phytopathogenic viral genera [4], among which the most important are the single-stranded DNA viruses of the genus *Begomovirus* (family *Geminiviridae*; [5]). Pakistan is a major global hotspot for begomovirus diseases, including cotton leaf curl disease (CLCuD), sustained despite long-term management. Indigenous *B. tabaci*, particularly Asia II-1, dominates across Pakistan, particularly in Punjab and northern regions, where it efficiently transmits CLCuD. It harbors greater begomovirus diversity than invasive MEAM1, which remains largely restricted to Sindh province in the southern region [6]. The predominance of Asia II-1 shows its adaptability to local climates and diverse hosts, with distinct genetic variants in virus transmission and insecticide resistance genes relative to MEAM1 [7]. Despite established distribution patterns, data linking endosymbiont compositions to virus transmission dynamics in Pakistan remain scarce. However, host genetics alone cannot explain persistent, regionally variable transmission, highlighting the role of microbial symbionts in vector competence.

Like other sap-feeding insects, *B. tabaci* harbors an obligate primary (P)-endosymbiont, *Candidatus Portiera aleyrodidarum*, which provides essential amino acids and vitamins deficient or absent in plant phloem sap [8]. This obligatory symbiosis is assumed to have co-evolved with their insect hosts for a long time [8,9] and has quite small genomes—smaller than 1000 kb and 600 genes. The synthesis of these essential nutrients requires metabolic complementation both from the host and the other endosymbionts [10]. While the nutritional contributions of P-endosymbionts are well-documented, the other important biological functions, such as their role in virus transmission, host fitness, and reproductive manipulation, remain less explored.

Beyond P-endosymbiont *Portiera*, *B. tabaci* variably harbor one or more facultative secondary (S)-endosymbionts, such as *Arsenophonus*, *Hamiltonella*, *Cardinium, Rickettsia*, and *Wolbachia*. These S-endosymbionts are transmitted both vertically and horizontally within the insect population [9,11,12]. These can profoundly influence host fitness, provide essential nutrients [13,14], resistance against parasitic wasps and fungal/viral pathogens [15,16,17,18,19,20], and stress and heat tolerance [21]. Conversely, certain S-endosymbionts, notably *Wolbachia*, *Arsenophonus*, *Cardinium*, and *Rickettsia*, act as reproductive manipulators by introducing cytoplasmic incompatibility, parthenogenesis, sex-ratio distortion, or male killing, thereby promoting their own spread to other hosts and ecological niches [22,23,24]. Beyond these effects, S-endosymbionts are increasingly recognized as key determinants of whitefly vector competence. In addition, they modulate virus acquisition, retention, and transmission [25] by providing thermotolerance [26] and resistance to parasitoids and various insecticides [27,28,29]. The *Candidatus Fritschea bemisiae*, an obligate intracellular endosymbiont harbored within bacteriocytes of the A biotype *B. tabaci* (USA), has been shown to have a role in reducing host fecundity and narrowing host range relative to *B. argentifolii*, though its precise role in begomovirus vector competence remains elusive [30].

The influence of S-endosymbionts on begomovirus transmission is rooted in specific molecular and physiological mechanisms. *Hamiltonella defensa*, *Rickettsia* spp., and *Arsenophonus* spp. have emerged as key modulators of this process. Notably, *Hamiltonella*-derived GroEL proteins protect tomato yellow leaf curl virus (TYLCV) within the insect vector [25], while *Hamiltonella*-associated modulation of odorant-binding proteins affects tomato chlorosis virus transmission [31,32]. Similarly, *Rickettsia* infections enhance virus retention, virus acquisition, and transmission efficiency [33]. Among these, *Arsenophonus* is a pivotal mediator of *B. tabaci* competence, forming stable, high-frequency associations with dominant cryptic species across Asia, the Middle East, and Africa [3,34,35]. Its mechanistic influence on begomovirus transmission is primarily driven by the expression of GroEL chaperonin proteins, which bind to coat proteins, protecting virions from degradation within the hemolymph and facilitating their circulation to the salivary glands [36]. Beyond direct viroprotection, *Arsenophonus* contributes to host nutritional supplementation and physiological homeostasis, potentially enhancing viral acquisition and retention [37,38]. Despite the importance of these mechanisms in driving persistent begomovirus epidemics, particularly in regions like Pakistan, dominated by the Asia II-1 cryptic species [39,40,41], the prevalence and epidemiological significance of *Arsenophonus* in local populations, particularly in Asia II 1, remains understudied.

Therefore, the present study investigates the occurrence and distribution of different endosymbionts, including *Arsenophonus*, *Porteira*, *Hamiltonella*, *Cardinium*, *Wolbachia*, *Rickettsia* and *Fritschea*, in the field populations of *B. tabaci* collected from major agricultural regions of Pakistan, including major cotton-growing areas. By integrating endosymbiont infection patterns, particularly *Arsenophonus*, with cryptic species composition, this study provides the first comprehensive assessment of the microbial ecology of Pakistani *B. tabaci* whiteflies and evaluates the potential role of *Arsenophonus* as a cryptic driver of begomovirus epidemiology. Ultimately, this research identifies an underappreciated biological lever in whitefly-mediated epidemics, offering new insights for the development of symbiont-targeted management strategies in South Asian agrosystems.

## 2. Materials and Methods

### 2.1. Whitefly Sampling and DNA Extraction

**Figure 1:**
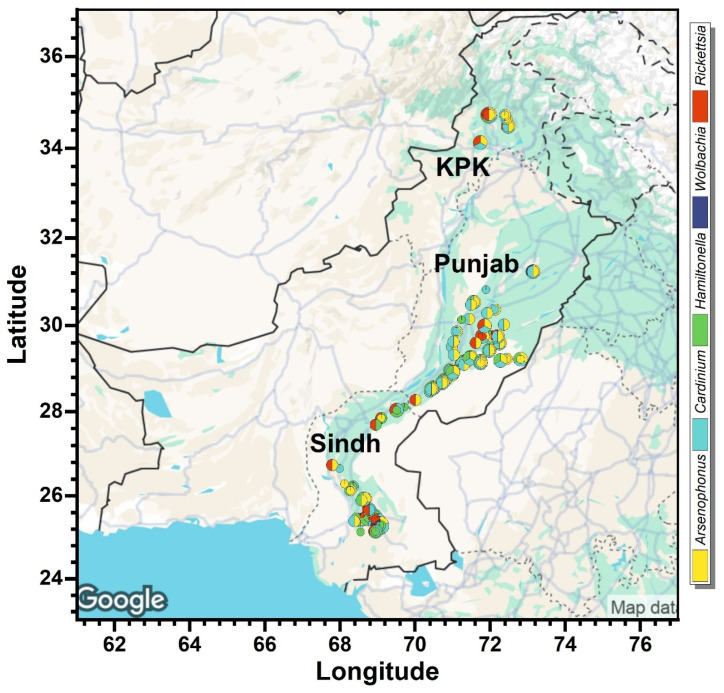
Spatial distribution of secondary endosymbionts in *B. tabaci* populations across Pakistan, including Punjab, Sindh, and Khyber Pakhtunkhwa (KPK). Each sampling location is represented by a pie chart positioned according to its geographic coordinates. The size, composition, and color of pies reflect the symbiont community structure of individual endosymbionts: *Rickettsia* (red), *Wolbachia* (blue), *Hamiltonella* (green), *Cardinium* (cyan), and *Arsenophonus* (yellow).

Adult *B. tabaci* were collected from various host plants, including cotton, chili, and okra, all showing leaf curl phenotype, as described earlier [6,40]. Sampling was conducted during 2012–2014 across major agricultural regions of Pakistan, with a primary focus on CLCuD-affected regions of Punjab, Sindh, and Khyber Pakhtunkhwa (KPK; Figure 1; Table S1). This targeted sampling strategy ensured representation from epidemiologically relevant hotspots of CLCuD, where disease pressure is consistently high and vector–virus dynamics are most pronounced. The GPS coordinates for each whitefly collection site were recorded using a handheld GPS device (eTrex 10; Garmin, Schaffhausen, Switzerland). The collected whitefly samples were kept in absolute ethanol at −80 °C until further processing. Total genomic DNA was extracted from a single whitefly and subjected to quantification and amplification of a 780 bp fragment of the mitochondrial cytochrome oxidase I (mtCOI-3ʹ) region, as previously described [40]. The resultant amplicons were sequenced (Macrogen, Seoul, Republic of Korea), and a total of 274 whiteflies were selected for downstream endosymbiont screening.

### 2.2. Molecular Identification of S-Endosymbionts

Each individual whitefly (*n* = 274) was screened for S-endosymbiont infection using species-specific PCR primers targeting the 16S ribosomal RNA (rRNA) gene for *Arsenophonus*, *Porteira*, *Hamiltonella*, *Cardinium*, *Wolbachia*, and *Rickettsia* and the 23S rRNA gene for *Arsenophonus* and *Fritschea* (Table 1). For *Arsenophonus*, initial amplification using 16S rDNA primers occasionally produced non-specific amplicons. Therefore, 23S rDNA primers were subsequently employed to improve detection specificity and ensure reliable identification. PCR was carried out along with all the necessary controls in a 10 µL reaction volume using 2X Green master mix (Thermo Fisher Scientific, Waltham, MA, USA) containing standard PCR reagents (dNTPs, Taq Buffer, MgCl_2_) and 2 µL of (3–6 ng.µL^−1^) template DNA. The resultant PCR products were resolved on agarose gel (1%), after staining with ethidium bromide, and visualized under UV illumination.

### 2.3. Cloning and Sequencing

The resultant PCR amplicons of each detected S-endosymbiont were purified and ligated into the pTZ57R/T vector (Thermo Fisher Scientific, USA). The resultant recombinant plasmids were transformed into competent *E. coli* cells (Top 10), and positive clones were sequenced by Sanger sequencing (at the USDA Mid-South Area Genomics Laboratory, Stoneville, MS, USA). Sequences were assembled and edited using the Lasergene software suite (DNAStar, Madison, WI, USA) and subsequently deposited in NCBI GenBank. The assigned accession numbers are listed in Table S1.

### 2.4. Phylogenetic Analysis

The nucleotide sequence alignments of all the S-endosymbionts, along with the NCBI-retrieved GenBank sequences, were used to infer phylogenetic relationships of each S-endosymbiont. The maximum likelihood (ML) phylogenetic trees employing the best-fit substitution model (according to the Bayesian information criteria [BIC]) were reconstructed using the IQTREE webserver (http://iqtree.cibiv.univie.ac.at/, accessed on 26 February 2026). The nodal support was evaluated by 1000 ultrafast bootstrap pseudoreplicates and the SH-aLRT branch test [47].

Subsequently, the resulting trees were visualized, edited, and appropriately re-rooted using related outgroup taxa, where applicable, in the FigTree v.1.4.2 (http://tree.bio.ed.ac.uk/software/figtree/, accessed on 2 March 2026).

### 2.5. Infection Frequency, Network- and Statistical Analysis

Infection frequencies of S-endosymbionts were estimated using binomial logistic regression. For each symbiont, infection status was modeled as the response variable, with *B. tabaci* cryptic species as categorical predictors. Predicted infection probabilities and standard errors were derived from fitted, generalized linear models (GLMs) with a binomial error distribution. Pairwise differences among cryptic species were evaluated using Tukey’s honestly significant difference (HSD) post hoc test with adjustment for multiple comparisons. All analyses were conducted in XLSTAT (Addinsoft, Paris, France), with significance set at *p* ≤ 0.05 and (k-1) degrees of freedom. To ensure clarity, statistical outcomes are reported based on pairwise comparisons and significance levels, without mentioning F-values.

Network analysis based on infection frequencies was performed in OriginPro v2024 using Pearson correlation coefficients calculated across S-endosymbiont presence in the collected *B. tabaci* populations. Pairwise associations (co-occurrence patterns) were inferred using Pearson correlation coefficients on binary presence–absence data, equivalent to the phi (φ) coefficient for dichotomous variables. All the figures were generated in OriginPro v2024 (OriginLab Corporation, Northampton, MA, USA).

## 3. Results

### 3.1. Identification of B. tabaci Species

Mitochondrial COI-based identification revealed the presence of six cryptic species of the *B. tabaci* complex in Pakistan, including Asia II-1, Asia II-5, Asia II-7, Asia II-8, Asia I and MEAM-1. Species identification was performed using the mtCOI-3 gene sequence through comparison with reference sequences available in the Genbank and phylogenetic analysis according to the established criteria for the *B. tabaci* species complex [2]. Among these, Asia II-1 prevailed in the Punjab region, while MEAM-1 was the most common biotype identified in Sindh [40]. Asia 1 was detected at low frequency, while Asia II-5, Asia II-7, and Asia II-8 were rare and represented by very few individuals.

### 3.2. Identification of Secondary Endosymbiont Infection

Of the six *B. tabaci* species identified in this study, Asia II-5 and Asia II-7 had just a few individuals and yielded no PCR amplification. Therefore, these species were excluded from the subsequent analyses. The remaining 274 adult whiteflies, representing four species, Asia II-1 (*n* = 199), MEAM-1 (*n* = 67), Asia 1 (*n* = 7), and Asia II-8 (*n* = 1), were screened for P- and S-endosymbionts (Table 1). The P-endosymbiont *Portiera* was detected in 100% of these whiteflies, confirming its obligate and ubiquitous association with *B. tabaci* populations in Pakistan.

Five S-endosymbionts, including *Arsenophonus*, *Cardinium*, *Hamiltonella*, *Rickettsia,* and *Wolbachia,* were identified in the *B. tabaci*, showing varying rates of infection frequency. Overall, 221 whiteflies (81%) harbored at least one S-endosymbiont, and multiple infections were common, with several whiteflies carrying up to four S-endosymbionts simultaneously. PCR amplification initially suggested the presence of *Fritschea*; however, sequencing of the amplicon revealed these to be false positives. This suggests that *B. tabaci* populations in Pakistan are not hosts to *Fritschea*.

### 3.3. Cryptic Species-Specific Infection Patterns

Marked differences in S-endosymbiont prevalence were observed among cryptic species. Of the five S-endosymbionts, four were present in the most prevalent biotype (cryptic species) Asia II-1; *Rickettsia* was identified but in only one sample. *Arsenophonus* was the most prevalent S-endosymbiont overall, detected in 68% (136/199) of Asia II-1 whiteflies, 21% (14/67) in MEAM-1, and 100% (7/7) in Asia 1. *Cardinium* was also widespread, with infection rates of 57% (4/7) in Asia 1, 37% (25/67) in MEAM-1, and 58% (117/199) in Asia II-1. *Hamiltonella* exhibited a contrasting distribution pattern, occurring at high frequency in MEAM-1 61% (41/67) but lower in Asia II-1 20% (39/199), and being undetected in Asia 1 (0/7). *Wolbachia* infection rate was 11% in Asia II-1 (22/199), 14% (1/7) in Asia 1 and 20% (14/67) in MEAM-1. *Rickettsia* was rare, detected in only a few whiteflies across all sampled populations.

### 3.4. Geographic Variation in Endosymbiont Prevalence

**Figure 2:**
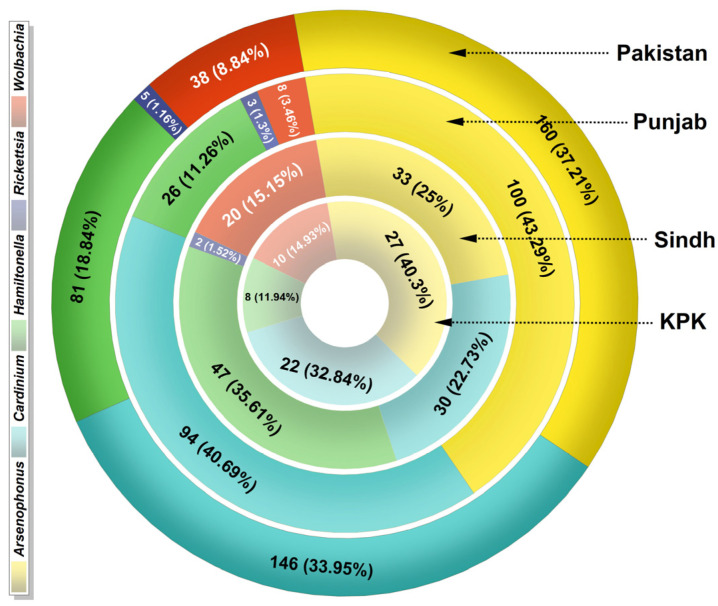
Regional prevalence of S-endosymbionts in Pakistani *B. tabaci* populations. Data are visualized using concentric donut charts: the outermost ring displays the aggregate national frequency, while inner rings represent provincial data for Punjab, Sindh, and Khyber Pakhtunkhwa (KPK). Color coding identifies specific endosymbionts: *Rickettsia* (blue), *Wolbachia* (red), *Hamiltonella* (green), *Cardinium* (cyan), and *Arsenophonus* (yellow). Segment labels provide both the raw number of infected individuals and their corresponding percentage within each region.

The distribution of S-endosymbionts varied significantly among the sampled whiteflies across Pakistan and three different provinces, including Punjab, Sindh, and KPK (Figure 2). At the national level, *Arsenophonus* was the most prevalent S-endosymbiont and was identified in 160 whiteflies (37%), followed by *Cardinium* in 147 (34%) and *Hamiltonella* in 82 (19%). While *Wolbachia* and *Rickettsia* were found in just 38 (8.8%) and 5 (1.2%) whiteflies, respectively.

Regional analysis revealed distinct variation in endosymbiont prevalence. In Punjab, *Arsenophonus* (*n* = 100, 43.3%) and *Cardinium* (*n* = 94, 40.7%) were the dominant endosymbionts. While *Wolbachia* (*n* = 8, 3.5%) and *Rickettsia* (*n* = 5, 1.24%) showed minimal presence. In contrast, the population in Sindh exhibited a higher prevalence of *Hamiltonella* (*n* = 47, 35.6%) compared to other regions, followed by *Arsenophonus* (*n* = 33, 25%), *Cardinium* (*n* = 30, 22.7%), and *Wolbachia* (*n* = 20, 15.26%). While *Rickettsia* (n = 2, 1.5%) was the least dominant in Sindh. In the KPK, *Arsenophonus* remained the most frequent endosymbiont (*n* = 27, 40.3%), followed by *Cardinium* (*n* = 23, 32.8%), while *Wolbachia* (*n* = 10, 14.9%) and *Hamiltonella* (*n* = 8, 11.9%) were present at lower frequencies. The results thus revealed that endosymbiont community composition is geographically structured and species-associated, with Punjab showing the highest overall diversity and Sindh exhibiting the strongest Asia II-1–*Arsenophonus* association.

### 3.5. Infection Frequency of S-Endosymbionts

**Figure 3:**
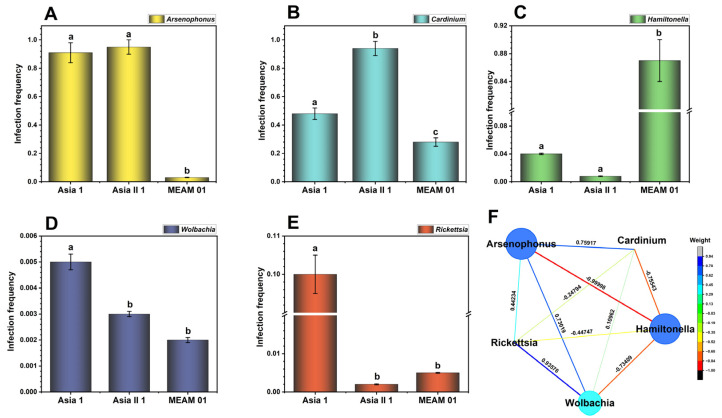
Infection frequency and co-occurrence network of S-endosymbionts across different *B. tabaci* cryptic species. (**A**–**E**) Prevalence and infection frequency of five S-endosymbionts—*Arsenophonus* (**A**), *Cardinium* (**B**), *Hamiltonella* (**C**), *Wolbachia* (**D**), and *Rickettsia* (**E**)—within the *B. tabaci* cryptic species Asia 1, Asia II-1, and MEAM 1. Vertical bars represent the mean infection frequency, and error bars indicate the standard error. Different lowercase letters above the bars denote statistically significant differences (*p* ≤ 0.05) based on post hoc testing. (**F**) Correlation-based network map illustrating the interactions between the endosymbiont communities. Nodes represent genera, with size indicating relative abundance and correlation strength. Blue and red lines represent positive and negative correlations, respectively, line thickness and numerical labels represent the weight of the interaction.

Infection frequency analysis showed distinctive cryptic species-associated patterns of S-endosymbiont occurrence in *B. tabaci* populations from Pakistan (Figure 3). *Arsenophonus* showed significantly (*p* ≤ 0.05) the highest infection frequency in dominant Asia II-1 and Asia 1 compared with all other S-endosymbionts. *Cardinium* also showed a moderate to high infection frequency of S-endosymbionts in Asia II-1 and Asia 1, while its probability was highest in MEAM-1 as compared to all other cryptic species. Whereas *Hamiltonella* and Wolbachia occurred at comparatively lower probabilities. In MEAM-1, infection frequency was dominated by *Hamiltonella*, which showed the highest likelihood of occurrence, followed by *Arsenophonus* and *Cardinium*. *Wolbachia* was present at a moderate frequency, while *Rickettsia* remained infrequent. Overall, these results demonstrate pronounced cryptic species-dependent variation in S-endosymbiont infection frequency.

The endosymbiont interaction network (Figure 3F) showed complex synergistic and antagonistic co-occurrence relationships between the endosymbiont communities. A strong synergistic association was identified between *Rickettsia* and *Wolbachia* (weight = 0.936) and *Arsenophonus* and *Cardinium* (0.759), suggesting frequent co-occurrence. Conversely, an antagonistic co-occurrence was inferred between *Arsenophonus* and *Hamiltonella* (−1.00), indicating a strong negative association, suggestive of competitive exclusion. *Hamiltonella* also showed negative correlations with *Cardinium* (−0.755) and *Wolbachia* (−0.734). These results suggest that while certain taxa readily co-infect the host, *Hamiltonella* acts as a primary competitor, significantly shaping the internal microbial community structure through niche partitioning or physiological incompatibility.

### 3.6. Phylogenetic Analysis of S-Endosymbionts

**Figure 4:**
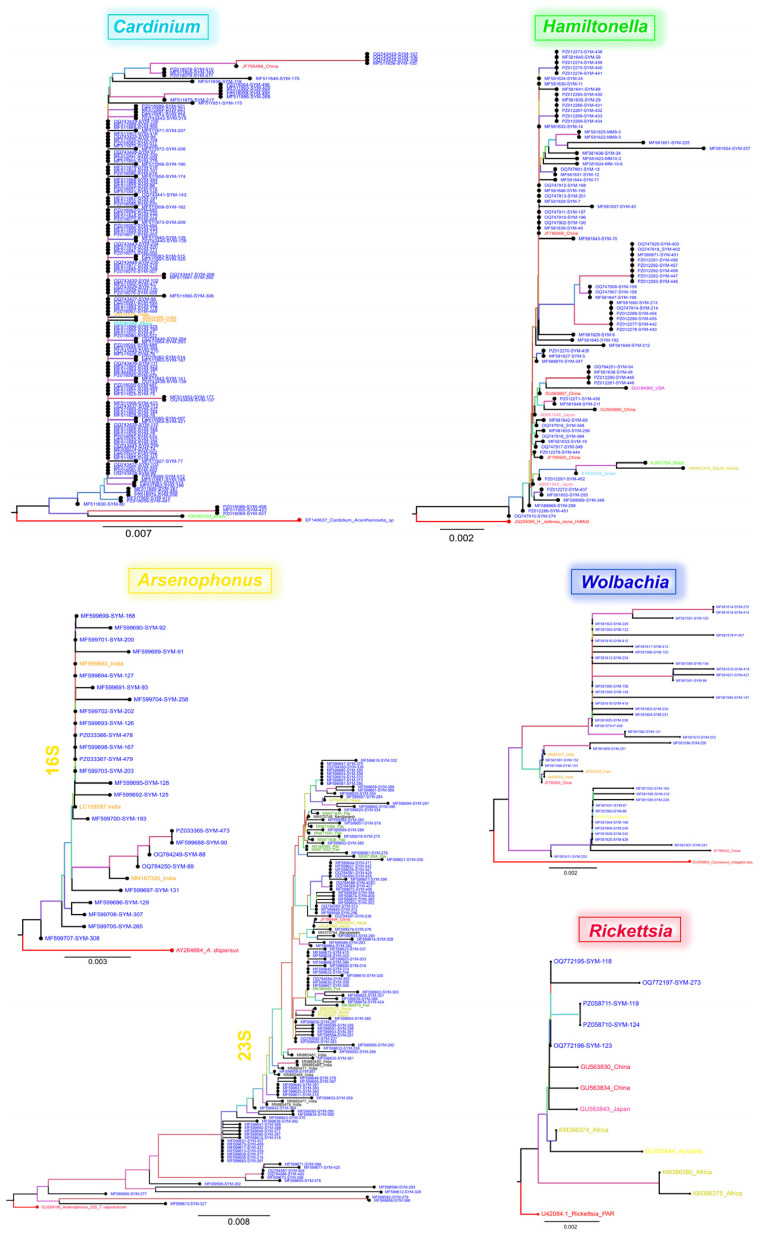
Phylogenetic analysis of S-endosymbionts identified in *B. tabaci*. Individual maximum-likelihood trees illustrate the evolutionary relationships and genetic diversity of *Arsenophonus*, *Cardinium*, *Hamiltonella*, *Wolbachia*, and *Rickettsia* based on representative marker sequences. All the trees were inferred with 1000 bootstrap values and all clades showed 50–100% bootstrap values. The best-fit models selected via BIC for each genus were: *Arsenophonus* (TPM3u + F [16S] and HKY + F + G4 [23S]), *Cardinium* (TPM2 + F), *Hamiltonella* (HKY + F), *Rickettsia* (HKY + F + I), and *Wolbachia* (K2P). Tip labels indicate sequence accessions, host information, or geographical origin (India [orange], China [red], Africa [green], Australia [yellow], USA [dark purple], Pakistan [dark green], Japan [pink], Nepal [brown], Bangladesh [light purple], and this study isolates in blue). All outgroup taxa are indicated by red lines and red font. Phylogenetic trees of all identified endosymbionts were constructed using 16S rDNA sequences, whereas the *Arsenophonus* phylogenies were inferred using both 16S and 23S rDNA sequences. Scale bars represent the number of nucleotide substitutions per site, and double slashes indicate shortened branches for visual clarity.

Phylogenetic analyses, based on 16S rRNA sequences for all endosymbionts and 16S and 23S rRNA for *Arsenophonus*, demonstrated that the Pakistani S-endosymbiont clustered with sequences reported from neighboring countries (Figure 4). *Cardinium* sequences from Asia II-1 and MEAM 1 obtained from Punjab, Sindh, and KPK grouped with *Cardinium* sequences reported from different species across different regions. While *Wolbachia* sequences clustered into three distinct groups, one of which showed resemblance to *Wolbachia* sequences obtained from India. This result demonstrated that *Wolbachia* may have three strains in Pakistani whiteflies. The *Hamiltonella* sequences from Asia II-1 and MEAM 1 were nearly identical; they clustered with the *Hamiltonella* sequences reported from MED and MEAM-1 biotypes in China. The *Arsenophonus* sequences, both 16S and 23S, from all three cryptic species, Asia II-1, Asia I, and MEAM-1, clustered into two major well-supported clades (Figure 4). Clade I, comprising all 23S rRNA sequences, formed a monophyletic group with sub-clusters corresponding to Pakistani, Indian, Bangladeshi, Nepali, and Chinese isolates, indicating geographic structuring. Clade II, comprising 16S rRNA sequences, grouped all the *Arsenophonus* isolates from this study with Indian isolates, supporting regional continuity of *Arsenophonus* strains among South Asian whitefly populations.

## 4. Discussion

The current study provides a comprehensive assessment of S-endosymbiont diversity and distribution in *B. tabaci* from CLCuD-affected regions of Pakistan. Our findings reveal a clear dominance of *Arsenophonus* in indigenous whitefly populations, particularly within the predominant Asia II-1 cryptic species. This contrasts with MEAM-1, which remained largely confined to the Sindh region. Although *Cardinium* shows comparable frequency, the emphasis on *Arsenophonus* is based on functional relevance rather than prevalence alone. *Arsenophonus* showed the most consistent distribution across cryptic species and regions, particularly virus-endemic areas. In contrast, *Cardinium* showed greater variability and was frequently co-infected with *Arsenophonus*, especially in Punjab. This pattern suggests that *Cardinium* may play a complementary or modulatory role, whereas *Arsenophonus* may be associated with virus transmission, consistent with its established role in virus transmission [48]. However, these associations remain observational and require functional validation.

Among the initially tested six S-endosymbionts, the primers for *Fritschea* gave false positive results, as verified by sequencing the product. So, it was concluded that Pakistani *B. tabaci* populations are not infected with the *Fritschea*. This aligns with global surveys in Israel [49], China [50], Croatia [35,51] and some European and African countries [52], where *Fritschea* was absent in *Bemisia* populations, remaining largely restricted to New World species in the USA [30,53].

The low prevalence of *Rickettsia* (detected in only five samples) suggests it is not a primary driver of whitefly fitness or reproductive manipulation in Pakistani populations, contrasting with the high abundance reported in neighboring India and China [36,54]. While *Rickettsia* is known as a reproductive manipulator [55,56], fertility booster [57], provision of heat tolerance [26], and enhancement of susceptibility to various chemical insecticides [28], its ecological significance in the Pakistani *Bemisia* population remains unclear. Its low presence may possibly be attributed to the difference in biotypes in these regions or specific ecological constraints. We anticipate that the dominant Pakistani biotype may possess a genetic background incompatible with *Rickettsia* colonization. Furthermore, while *Rickettsia* can confer heat tolerance in temperate climates, the extreme temperatures of the Pakistani agro-ecosystem, often exceeding 45 °C, may render the metabolic cost of maintaining the symbiont higher than its protective value. Additionally, competitive exclusion by S-endosymbionts, such as *Arsenophonus*, may prevent *Rickettsia* from achieving a stable foothold. Consequently, the interaction of host genetics, extreme heat, and microbial competition likely exerts selective pressure that maintains *Rickettsia* at near-incidental levels within these populations.

In the current study, *Hamiltonella* was detected in all samples of MEAM-1 biotypes collected from Sindh, Pakistan. This finding aligns with previous reports [49,54], confirming the high prevalence of this S-endosymbiont in MEAM-1. Importantly, we also identified a small number of Asia II-1 samples positive for *Hamiltonella,* while there have been no global reports documenting the presence of Asia II-1 positive for *Hamiltonella*. The presence of *Hamiltonella* is in a few Asia II-1 samples, maybe due to the horizontal transfer among different insects. Whether such low-frequency infections translate into functional effects on virus transmission remains unknown but warrants targeted experimental investigation. Nonetheless, a previous study indicated that GroEL proteins produced by *Hamiltonella* present in the MEAM 1 population are known to facilitate TYLCV transmission [25]. This is further supported by observations in Israel, where MED populations, which do not harbor *Hamiltonella*, exhibit poor TYLCV transmission efficiency. Conversely, in China, both MEAM1 and MED populations harbor *Hamiltonella* at high incidences [50,54] and are highly efficient vectors of TYLCV [58]. However, native Asia II-1 does not harbor *Hamiltonella*, but still, it remains highly capable of transmitting several begomoviruses, including TYLCV and cotton leaf curl Multan virus (CLCuMuV) with high efficiency [39,48,58,59,60]. This suggests that Asia II-1 may rely on alternative mechanisms or different endosymbionts to achieve high transmission efficiency.

*Arsenophonus* was by far the most prevalent, especially in Asia II-1 and Asia I populations, and exhibited pronounced geographic structuring, with the highest frequencies in Punjab and KPK regions characterized by intense and persistent begomovirus pressure. Our finding coincides with earlier reports, which reported the presence of *Arsenophonus* in different whitefly species in different regions of the world [36,49,52,54]. In this study, *Arsenophonus* infection was found in MEAM 1 but with a very low rate. Previous studies have suggested potential molecular interactions between *Arsenophonus* and virus components, including reported binding of bacterial GroEL with viral coat proteins of CLCuD-associated begomoviruses (possibly CLCuMuV), which may influence virus retention or stability in the insect vector. In addition, *Arsenophonus* has been detected in multiple whitefly tissues, including bacteriocytes, salivary glands, and the midgut of *Bemisia tabaci* [36]. It has been revealed that Asia II-1 transmits CLCuMuV efficiently [48], and the occurrence of *Arsenophonus* in Asia II-1 suggests a potential association in virus transmission in the Pakistani Asia II-1 population which needs to be investigated. The high prevalence of *Arsenophonus* in dominant Pakistani whitefly populations therefore may represent a microbial factor contributing to the efficiency and durability of begomovirus transmission in the region.

While *Wolbachia* was identified across all tested biotypes, including Asia II-1, MEAM-1, and Asia 1, its infection frequency was notably lower in MEAM 1 and Asia 1. This suggests that in the Pakistani *Bemisia* population, *Wolbachia* may not be actively involved in manipulating reproduction or sex ratios, a role it commonly plays in many arthropods. Typically, *Wolbachia* acts as a reproductive manipulator by inducing cytoplasmic incompatibility, parthenogenesis, and killing males [61,62]. However, *Wolbachia* presence varies globally; earlier reports showed it was restricted to the Q biotype/species but not in the B biotype/species in Israeli populations [49]. Similarly, ref. [52] reported the *Wolbachia* presence in Q1 and Q2 but not in B and Q3. However, in Chinese *B. tabaci* populations, *Wolbachia* infection occurs at a high rate in B and Q biotypes/species and indigenous biotypes/species [63,64]. However, no reproductive or fitness-related traits were examined in the present study; therefore, any functional interpretation remains speculative. Low *Wolbachia* prevalence across all cryptic species may suggest that reproductive manipulation is not a primary driver of whitefly population structure in Pakistan.

The high infection rate of *Cardinium* in Asia II-1, often occurring as a co-infection with *Arsenophonus* in high viral incidence areas, points towards a potential synergistic effect on host biology. *Cardinium* is vastly present in several insects and arthropods like mites, ticks, spiders, spider mites, and copepods and approximately 7% of all arthropod species are infected with members of this genus [65,66,67]. *Cardinium hertigii*, for the first time, was detected in the *Encarsia* wasp, which parasitoids *B. tabaci* [65]. In many arthropods, *C. hertigii* has been defined to induce feminization, cytoplasmic incompatibility, and parthenogenesis [68]. While *Cardinium* is often considered mutualistic in *Bemisia,* a recent report [69] showed that *Cardinium* reduced its genome size to improve its survival within the *B. tabaci*. As per previous studies, the infection of *Cardinium* in MEAM-1 and MED was very low as compared to the native whiteflies [50,52,70]. In the current study, *Cardinium* was detected in all three species, albeit with variable infection frequency. Notably, the infection rate was highest in Asia II-1. In the Punjab region—an area characterized by high viral incidence—*Cardinium* was often found in co-infection with *Arsenophonus*. Specifically, within Punjab populations, *Arsenophonus* (43.1%) and *Cardinium* (40.5%) exhibited the highest regional prevalence. This co-occurrence in Asia II-1 suggests that the presence of *Cardinium* may exert an influence on host biology, potentially modulating fitness or whitefly virus interactions under these specific agro-ecological stresses. The heavy pesticide use in this region may further select for symbiont combinations that enhance host detoxification or survival, reinforcing the dominance of these specific lineages.

Phylogenetic analyses revealed that Pakistani symbiont strains cluster with those from neighboring countries, indicating regional continuity rather than the emergence of highly divergent local lineages [71]. This finding suggests that ecological factors, host species composition, and symbiont prevalence—rather than strain novelty—are likely the primary contributors shaping vector–virus dynamics in Pakistan.

This study offers valuable insights into the genetic landscape of cryptic species and their endosymbionts yet has key limitations. First, samples collected between 2012 and 2014 provide a critical historical baseline but may not reflect current evolutionary dynamics or recent population shifts. Second, reliance on PCR-based sequencing, while effective for known lineages, may miss highly divergent cryptic symbionts detectable only via unbiased high-throughput sequencing. Third, findings are based on molecular and phylogenetic associations without functional validation. Nonetheless, this work establishes a foundational framework to guide future experimental validation and long-term epidemiological surveillance in the region.

## 5. Conclusions

This study establishes that Pakistani *B. tabaci* populations function as a structured microbial holobiont, with *Arsenophonus* and *Cardinium* as dominant components. Their high prevalence, particularly in Asia II-1, suggests a possible potential interaction between symbiont composition, vector population and begomovirus epidemiology; the findings remain correlative. These results provide a strong foundation for understanding the host-symbiont associations in Pakistan and require further functional and mechanistic studies to clarify the role of symbionts in virus transmission and vector biology.

## Figures and Tables

**Table 1:** Primers and PCR conditions used in the analysis of endosymbiont diversity.

Target Gene	Primer Name	Primer Sequence (5’−3’)	Annealing Temperature (°C)	Amplicon Size (bp)	Reference
*B. tabaci* mtCOI	C1-J-2195 L2-N-3014	TTGATTTTTTGGTCATCCAGAAGT TCCAATGCACTAATCTGCCATATTA	45	~866	[42]
Portiera 16S rDNA	28F 1495R	TGCAAGTCGAGCGGCATCAT CTACGGCTACCTTGTTACGA	60	~1500	[43]
Arsenophonus 23S rDNA	Ars-23S-1 Ars-23S-2	CGTTTGATGAATTCATAGTCAAA GGTCCTCCAGTTAGTGTTACCCAAC	60	~600	[34]
Arsenophonus 16S rDNA	92-F 1343-R	TGAGTAAAGTCTGGGAATCTGG CCCGGGAACGTATTCACCGTAG	58	~1250	[43]
Cardinium 16S rDNA	CFB-F CFB-R	GCGGTGTAAAATGAGCTTG ACCTCTTCTTTAACTCAAGCCT	58	~400	[44]
Fritschea 23 rDNA	U23GISR 23GISR	GATGCCTTGGCATTGATAGGCGATGAAGGA TGGCTCATCATGCAAAAGGCA	60	~600	[30]
Hamiltonella 16S rDNA	Ham-F Ham-R	TGAGTAAAGTCTGGGAATCTGG AGTTCAAGACCGCAACCTC	60	~700	[43]
Rickettsia 16S rDNA	Rb-F Rb-R	GCTCAGAACGAACGCTATC GAAGGAAAGCATCTCTGC	60	~900	[45]
Wolbachia 16S rDNA	Wol16S-F Wol16S-R	CGGGGGAAAAATTTATTGCT AGCTGTAATACAGAAAGTAAA	55	~700	[46]

## Data Availability

The datasets generated and/or analyzed during the current study are available in the Gen bank INSDC member repository. Accessions for the *Arsenophonus*: PZ044229-PZ044252, MF599578-MF599707, OQ784249- OQ784250, OQ784581-OQ784591. Accessions for the *Cardinium*: PZ018054-PZ018094, MF464655, MF509274, MF511825-MF511906, OQ743424-OQ743448. Accessions of *Hamiltonella*: MF581627-MF581654, MF588868-MF588871, OQ747901-OQ747920, OQ784251, PZ012265-PZ012293. Accessions of *Wolbachia*: MF581581-MF581592, MF581594-MF581596, MF581598, MF581600-MF581612, MF581614-MF581619-MF581621, MF581578-MF581579. Accessions of *Rickettsia*: OQ772195-OQ772197, PZ058710, PZ05815711.

## Supplementary Materials

The following supporting information can be downloaded at: https://www.mdpi.com/article/10.3390/insects17060585/s1, Table S1: Whitefly collection, their species, and endosymbionts details, including their collection sites, accession numbers, and host.

## Abbreviations

The following abbreviations are used in this manuscript:

MEAM 1	Middle East Asia Minor 1
MED	Mediterranean
TYLCV	tomato Yellow Leaf Curl Virus
CLCuMuV	Cotton leaf curl Multan virus
