# Supplementary material for: Prevalence and Distribution of Endosymbionts in Bemisia tabaci Populations from Pakistan: Dominance of Arsenophonus in Indigenous Asia II-1 Population
Source: Insects. 2026 Jun 3;17(6):585. doi: 10.3390/insects17060585 (PMC13300422; doi:10.3390/insects17060585)
Supplement: Supplementary file 1 [file insects-17-00585-s001.zip › insects-4264314-supplementary.pdf]

# - Supplementary data –

Table S1. Whitefly collection, their biotype, and endosymbionts details, including their collection sites, accession numbers, and host.

Sr No	Collection sites	COI Accessions	Province	biotype	hosts	Symbionts				
						A	C	H	R	W
1	Rasidabad, TA yar	HG918195	S	Asia II-1	Cotton	MF599649	MF511840	-	-	-
2		HG918196	S	Asia I		MF599650	OQ743445	-	-	-
3		HG918197	S	Asia II-1		MF599651	OQ743440			
4		HG918198	S	MEAM-1		MF599652	-	MF581635	-	-
5		OM256501	S	Asia II-1		-	-	-	-	MF581584
6	ARI, Tandojam	LN897433	S	MEAM-1	Cotton	MF599648	MF511874	PZ012265	-	-
7		OM256502	S	MEAM-1		MF599635	MF511860	PZ012266	-	-
8		OM256503	S	MEAM-1		MF599653	MF511838	PZ012267	-	-
9		OM256504	S	MEAM-1		MF599657	MF511839	PZ012268	-	-
10		OM256505		MEAM-1		MF599658	-	-	-	-
11	Tandoo Allah yar	HG918199	S	MEAM-1	chillies	MF599653	MF511878	PZ012269	-	MF581614
12		HG918200	S	MEAM-1		-	-	-	-	-
13		HG918201	S	MEAM-1		-	-	MF581627	-	-
14		HG918202	S	MEAM-1		-	-	MF581636	-	-
15		OM256506	S	MEAM-1		-	-	-	-	-
16	Mirpur khas	HG918203	S	MEAM-1	Cotton	-	OQ743447	MF581628	-	MF581605
17		HG918204	S	MEAM-1		-	-	-	-	-
18		HG918205	S	MEAM-1		-	-	MF581629	-	-
19		OM256507	S	MEAM-1		-	-	OQ747901	-	-
20		OM256508	S	MEAM-1		-	-	OQ747914	-	MF581578
21	Khurra goth, Mirwah	HG918206	S	MEAM-1	Okra	-	OQ743438	MF581630	-	MF581585
22		OM256509	S	MEAM-1		-	-	MF581631	-	-
23		OM256510	S	MEAM-1		-	MF511842	-	-	-

24		OM256511	S	MEAM-1		-	-	-	-	-
25		OM256512	S	MEAM-1		-	-	-	-	-
26	Mirwah	HG918207	S	MEAM1	Cotton	MF599654	MF511843	PZ012270	OQ772195	MF581596
27		LN897434	S	MEAM1		MF599655	OQ743441	-	-	-
28		OM256513	S	Asia II-1		-	-	MF581648	OQ772196	-
29		OM256514	S	Asia II-1		-	-	MF581651	-	-
30		OM256515	S	Asia II-1		-	-	-	-	-
31	Tando Ghulam Ali	HG918208	S	MEAM-1	Cotton	-	OQ743439	MF581649	-	OQ747924
32		HG918212	S	MEAM-1		-	-	MF581632	-	MF581596
33		HG918213	S	MEAM-1		-	MF511853	OQ747916	-	-
34		OM256516	S	MEAM-1		-	MF511875	-	-	-
35		OM256517	S	MEAM-1		-	-	OQ747917	-	-
36	Maatli	HG918214	S	MEAM-1	Cotton	-	MF511876	PZ012271	-	MF581597
37		HG918215	S	MEAM-1		-	MF511877	-	-	-
38		HG918183	S	MEAM-1		-	OQ743443	MF581652	-	-
39		OM256518	S	MEAM-1		-	PZ018054	MF581654	-	MF581598
40		OM256518	S	MEAM-1		-	PZ018055	-	-	MF581609
41	Tando Muhammad Khan	HG918209	S	MEAM-1	Cotton	-	-	OQ784251	-	-
42		LN832283	S	MEAM-1		-	-	PZ012272	-	-
43		OM256520	S	MEAM-1		-	-	PZ012273	-	-
44		OM256521	S	MEAM-1		-	-	PZ012274	-	-
45		OM256522	S	MEAM-1		-	-	PZ012275	-	-
46	Goth Muktar Arain	LN832284	S	MEAM 1	Cotton	-	MF511881	-	-	-
47		OM268948	S	MEAM 1		-	-	-	-	-
48		OM268949	S	MEAM 1		-	-	-	-	-
49		OM268950	S	MEAM 1		-	-	-	-	-
50		OM268951	S	Asia II-1		-	-	-	-	-
51	Chalgri, Hyderabad	LN832285	S	MEAM 1	Cotton	-	-	PZ012276	-	-
52		OM268952	S	MEAM 1		-	-	-	-	-
53		OM268953	S	MEAM 1		-	-	MF581650	-	-
54		OM268954	S	MEAM 1		-	-	MF581653	-	-
55		OM268955	S	MEAM 1		-	-	-	-	-

56	Goth Ali Khan Baloch	LN832286	S	Asia II-1	Cotton	MF599656	-	-	-	MF581586
57		LN832287	S	Asia II-1		MF599664	-	-	-	-
58		OM268956	S	Asia II-1		-	-	-	-	MF581603
59		OM268957	S	Asia II-1		MF599665	-	-	-	-
60		OM268958	S	Asia II-1		-	-	-	-	MF581599
61	Shadadpur	LN832288	S	MEAM 1	Cotton	MF599597	MF511863	MF581639	-	-
62		LN897437	S	Asia II-1		-	-	-	-	-
63		LN832290	S	Asia II-1		-	-	-	-	-
64		LN832291	S	MEAM 1		-	-	-	-	-
65	Jhandoolo shah	LN832293	S	Asia II-1	Cotton	MF599598	-	PZ012277	-	-
66		LN832293	S	Asia II-1		-	-	-	-	-
67		LN832294	S	Asia II-1			-	-	-	-
68		OM268959	S	Asia II-1		-	-	-	-	-
69		OM268960	S	Asia II-1		-	-	-	-	-
70	Nawab Shah	LN832295	S	MEAM 1	Cotton	-	MF511854	MF581645	-	-
71		LN832296	S	Asia II-1		-	-	MF588869	-	-
72		OM268961	S	Asia II-1		-	-	-	-	-
73		OM268962	S	Asia II-1		-	-	-	-	-
74	Moro	LN832307	S	Asia II-1	Cotton	-	MF511867	-	-	-
75		LN832308	S	MEAM 1		-	-	-	-	-
76		LN832311	S	MEAM 1		-	-	-	-	-
77		OM268963	S	MEAM 1		-	-	-	-	-
78		OM268964	S	MEAM 1		-	-	-	-	-
79	3km Moro	LN832297	S	Asia II-1	Cotton	MF599618	-	MF588870	-	-
80		LN832298	S	Asia II-1		MF599619	-	PZ012278	-	-
81		OM268965	S	Asia II-1		-	-	-	-	-
82		OM268966	S	Asia II-1		-	-	MF581637	-	-
83		OM268967	S	Asia II-1		-	-	-	-	-
84	Sakrand	LN832299	S	Asia II-1	Cotton	MF599636	-	-	-	-
85		LN832289	S	Asia II-1		-	-	-	-	-
86		LN832303	S	Asia II-1		MF599700	-	-	-	-
87		LN832304	S	Asia II-1		-	-	-	-	-

88		LN832305	S	Asia II-1		-	-	-	-	-
89	Qazi abad	LN832306	S	Asia II-1	Cotton	MF599616	-	-	-	-
90		OM268968	S	Asia II-1		-	-	-	-	-
91		OM268969	S	Asia II-1		MF599617	-	-	-	-
92		OM268970	S	Asia II-1		MF599663	-	-	-	-
93		OM268971	S	Asia II-1		-	-	-	-	-
94	Dadoo	LN832312	S	Asia II-1	Cotton	-	-	-	-	-
95		LN832309	S	Asia II-1		MF599599	-	-	-	-
96		LN832310	S	Asia II-1		MF599600	-	-	-	MF581581
97		OM268972	S	Asia II-1		-	-	-	-	-
98		OM268973	S	Asia II-1		-	-	-	-	-
99	Ratha abad (Abaru)	LN835373	S	MEAM-1	Cotton	-	-	MF581638	-	-
100		LN835374	S	MEAM-1		-	-	-	-	-
101		LN835375	S	MEAM-1		-	-	PZ012279	-	-
102		LN835376	S	MEAM-1		-	-	-	-	-
103	Pano Aqil	LN835378	S	MEAM 1	Cotton	-	-	OQ747909	-	OQ747923
104		LN835379	S	Asia II-1		-	-	OQ747911	-	MF581616
105		LN835380	S	Asia II-1		MF599636	-	-	-	-
106	Marot	LN835395	S	Asia II-1		-	PZ018056	-	-	-
107		LN835396	S	Asia II-1		MF599660	PZ018057	-	-	-
108		LN835397	S	Asia II-1		MF599661	PZ018058	-	-	-
109		LN835398	S	Asia II-1		MF599662	-	-	-	-
110	Mirpur Methalo	LN835377	S	MEAM 1		-	-	-	-	MF581611
111		LN835385	S	MEAM 1		-	MF511885	MF581640	-	MF581604
112		LN835386	S	MEAM 1		-	-	MF581643	-	-
113	Hyderabad		S	Asia 1	Cotton	MF599631	MF511859	-	-	-
114	Mandu dhero	LN835387	S	MEAM 1	cotton	-	-	MF581646	-	
115		LN835399	S	Asia II-1		-	-	-	-	-
116	Bunar	HG918192	K	Asia II-1	Okra	MF599592	MF511845	-	-	MF581606
117		HF935001	K	Asia II-1		MF599593	MF511846	-	-	-
118		HF935002	K	Asia II-1		-	-	-	-	-
119		OM098435	K	Asia II-1		-	-	-	-	-
120	Swat	LN897430	K	Asia II-1	Okra	MF599580	MF511847	-	-	-

121		OM098436	K	Asia II-1		MF599581	-	-	-	-
122		OM098437	K	Asia II-1		MF599582	MF511848	-	-	-
123		OM098438	K	Asia II-1		MF599583	-	-	-	-
124	Mingora	LN897455	K	Asia II-1	Okra	MF599594	MF511857	-	-	-
125		OM098439	K	Asia II-1		MF599595	-	-	-	-
126		OM098440	K	Asia II-1		MF599596	MF511858	-	-	-
127		OM098441	K	Asia II-1		MF599620	-	-	-	-
128		OM098442	K	Asia II-1		MF599637	-	-	-	-
129	Osakay	HG918210	K	Asia II-1	Cucumber	MF599578	MF511849	OQ747915	-	-
130		HG918211	K	Asia II-1		MF599579	MF511850	OQ747907	-	-
131		LN897435	K	Asia II-1		MF599612	MF511851	MF581641	-	MF581589
132		LN897436	K	Asia II-1		MF599613	-	MF581642	-	OQ747925
133		OM098443	K	Asia II-1		-	MF511852	-	-	-
134		OM098444	K	Asia II-1		MF599614	MF511868	OQ747908	-	MF581590
135		OM098445	K	Asia II-1		-	MF511869	-	-	-
136		OM098446	K	Asia II-1		MF599615	MF511870	MF581633	-	
137		OM098447	K	Asia II-1		MF599584	MF511871	-	-	OQ747926
138		OM098448	K	Asia II-1		MF599585	MF511872	-	-	-
139		OM098449	K	Asia II-1		MF599586	MF511873	MF581634	-	-
140		OM098450	K	Asia II-1		MF599587	MF511879	-	-	-
141		OM098451	K	Asia II-1		MF599588	MF511880	MF581644	-	-
142		OM098452	K	Asia II-1		MF599589	OQ743446	-	-	MF581591
143		OM098453	K	Asia II-1		MF599590	MF511883	-	-	-
144		OM098454	K	Asia II-1		MF599591	MF511884	-	-	MF581592

145		OM098455	K	Asia II-1		MF599592	-	-	-	MF581593
146	Charsadda	LN897451	K	Asia II-1	Weeds	MF599593	MF511847	-	-	MF581594
147		LN897452	K	Asia II-1		-	-	-	-	-
148		OM098456	K	Asia II-1		-	-	-	-	-
149		OM098457	K	Asia II-1		-	-	-	-	-
150		OM098458	K	Asia II-1		-	-	-	-	-
151	Faisalabad	HF934975	P	Asia II-1	Cotton	MF599602	MF511832	-	OQ772197	-
152		HF934976	P	Asia II-1		MF599603	MF511833	-	-	-
153		HF934977	P	Asia II-1		MF599604	-	-	PZ058710	-
154		HG918194	P	Asia II-1			PZ018073	-	PZ058711	-
155		OM131699	P	Asia II-1		OQ784582	PZ018074	-	-	-
156	Kaccha khu	HF934982	P	Asia II-1	Cotton	MF599666	MF511900	-	-	-
157		OM131700	P	Asia II-1		MF599667	MF511901	-	-	-
158		OM131701	P	Asia II-1		-	MF511902	-	-	-
159		OM131702	P	Asia II-1		OQ784581	PZ018059	-	-	-
160		OM131703	P	Asia II-1		-	PZ018060	-	-	-
161	Khanewal	HF934983	P	Asia II-1	Cotton	MF599668	PZ018061	-	-	-
162		OM131704	P	Asia II-1		MF599669	PZ018062	-	-	-
163		OM131705	P	Asia II-1		MF599676	PZ018063	-	-	-
164		OM131706	P	Asia II-1		PZ044229	PZ018064	-	-	-
165		OM131707	P	Asia II-1		-	-	-	-	-
166	Bahawalpur	HF934986	P	Asia II-1	Cotton	PZ044230	PZ018065	PZ012290	-	-
167		OM131708	P	Asia II-1		PZ044231	-	-	-	-
168		OM131709	P	Asia II-1		PZ044232	PZ018066	PZ012291	-	-
169	Wahi Hussain	HF934987	P	Asia 1	Brinjal	MF599670	PZ018067	-	-	MF581615
170		HF934997	P	Asia 1		MF599671	PZ018068	-	-	-
171		HG315652	P	Asia 1		OQ784586	-	-	-	-
172		HG315653	P	Asia 1		MF599630	-	-	-	-
173		HG315654	P	Asia 1		MF599699	-	-	-	-

174	Rahim Yar Khan	LT222297	P	Asia II-1	Cotton	OQ784588	MF511904	-	-	-
175		HG315647	P	Asia II-1		MF599672		-	-	-
176		HG315648	P	Asia II-1		OQ784589	MF511886	-	-	-
177		LN835382	P	Asia II-1		MF599673	PZ018069	-	-	-
178		LN835383	P	Asia II-1		PZ044233	PZ018070	-	-	-
179		LN835384	P	Asia II-1		PZ044234	PZ018071	-	-	-
180		OM281714	P	Asia II-1		PZ044235	PZ018072	-	-	-
181	Kot Samaba	HF934993	P	Asia II-1	Cotton	MF599674	MF511905	MF588871	-	-
182		HG315657	P	Asia II-1		MF599675	MF511906	-	-	-
183		HG315658	P	Asia II-1		PZ044236	MF511882	-	-	-
184	Firoza	HF934992	P	Asia II-1		PZ044237	PZ018075	OQ747904	-	-
185		HF935005	P	Asia II-1		PZ044238	PZ018076	-	-	-
186		HG315649	P	Asia II-1		PZ044239	PZ018077	-	-	-
187		HG315650	P	Asia II-1			OQ743425	-	-	-
188	CCRI Multan	LN897423	P	Asia II-1	Cotton	PZ044240	PZ018078	-	-	-
189		OM131710	P	Asia II-1		PZ044241	-	-	-	-
190		OM131711	P	Asia II-1		PZ044242	PZ018079	-	-	-
191	Kot bahadar	LN832300	P	Asia II-1	Caster beans	-	-	-	-	-
192		LN832320	P	Asia II-1		-	-	-	-	-
193	Garh Maharaja	LN832301	P	Asia II-1	Cotton	-	MF464655	-	-	-
194		LN832302	P	Asia II-1		-	OQ743424	-	-	-
195	Rangpur	LN832313	P	Asia II-1	Cotton	OQ784249	MF509274	PZ012293	-	-
196		LN832314	P	Asia II-1		MF599698	MF511825	-	-	-
197		LN832315	P	Asia II-1		-	-	-	-	-

198	Rangpur (desert)	LN832318	P	Asia II-1	Cotton	MF599704	MF511826	-	-	-
199		LN832319	P	Asia II-1		OQ784250	MF511827	-	-	-
200	Khangarh (pathan wala)	LN832326	P	Asia II-1	Cotton	PZ033365	MF511828	-	-	-
201		OM131712	P	Asia II-1		-	MF511829	-	-	-
202	Hamza wali	LN832332	P	Asia II-1	Cotton	MF599690	MF511855	-	-	-
203		OM131713	P	Asia II-1		MF599628	MF511886	-	-	-
204	Ahmad Pur east	LN832357	P	Asia II-1	Cotton	MF599640	MF511834	OQ747912	-	-
205		LN832358	P	Asia II-1		MF599641	OQ743430	OQ747913	-	-
206		LN832334	P	Asia II-1		MF599642	OQ743431	-	-	-
207	Syed shahi wala	LN832335	P	Asia II-1	Cotton	MF599643	MF511835	-	-	-
208		LN832336	P	Asia II-1		MF599644	MF511836	-	-	-
209		LN832359	P	Asia II-1		PZ044243	OQ743432	-	-	-
210	Sui Vayar	LN832337	P	Asia II-1	Cotton	MF599692	MF511861	OQ747902	-	-
211		LN832348	P	Asia II-1		OQ784584	MF511864	PZ012281	-	-
212	Liaqatpur	LN832338	P	Asia II-1	Cotton	MF599638	OQ743427	PZ012282	-	-
213		LN832339	P	Asia II-1		MF599639	OQ743428	-	-	-
214		LN832340	P	Asia II-1		MF599659	OQ743429	-	-	-
215	Qila derawar	LN832341	P	MEAM-1	Cucur bits	MF599645	OQ743433	-	-	-
216		LN832342	P	MEAM-1		PZ044244	PZ018080	-	-	-
217		LN897438	P	Asia II-1		PZ044245	PZ018081	-	-	-

218	Rohi desert	LN832343	P	MEAM-1	Cotton	OQ784581	MF511887	MF588868	-	-
219		LN832344	P	MEAM-1		OQ784582	-	PZ012292	-	-
220		OM131714	P	Asia II-1		PZ044246	OQ743448	PZ012289	-	-
221	42 Adda	LN832345	P	Asia II-1	Cotton	OQ784585	MF511891	-	-	-
222	Desert near Fort Abbas	LN897439	P	Asia II-1	Cotton	MF599646	OQ743434	PZ012283	-	
223		LN832346	P	Asia II 8		MF599703	PZ018083	MF581647	-	-
224		LN832347	P	Asia II-1		-	-	-	-	-
225	Fort Abbas	LN835360	P	Asia II-1	Cotton	PZ033366	MF511895	PZ012284	-	-
226		LN835361	P	Asia II-1		-	-	-	-	-
227		LN835366	P	Asia II-1		PZ033367	-	-	-	-
228	Jetha Bhutta	LN832350	P	Asia II-1	Cotton	MF599693	MF511862	PZ012285	-	-
229		LN832351	P	Asia II-1		MF599697	MF511865	-	-	-
230	Kahror pakka	LN897453	P	Asia II-1	Cotton	MF599626	MF511893	PZ012286	-	-
231		OM281715	P	Asia II-1		MF599696	MF511894	OQ747920	-	-
232		OM281716	P	Asia II-1		MF599702	-	-	-	-
233		OM281717	P	Asia II-1		-	-	-	-	-
234	Muhammad Nagar	LN83252	P	Asia II-1	Cotton	MF599632	PZ018082	PZ012287	-	-
235		LN832353	P	Asia II-1		OQ784590	MF511856	OQ747903	-	-
236		LN832354	P	Asia II-1		MF599694	OQ743444	-	-	-
237	Duniya pur	OM131715	P	Asia II-1	Chillies	MF599706	MF511896	-	-	MF581619
238		OM131716	P	Asia II-1		-	-	-	-	-
239		OM131717	P	Asia II-1		-	-	-	-	MF581582
240		OM131718	P	Asia II-1		-	-	-	-	-
241	Lodhran	HF935006	P	Asia II-1	Cotton	MF599601	-	-	-	MF581620
242		OM281718	P	Asia II-1		-	-	-	-	-

243		OM281719	P	Asia II-1		-	-	-	-	-
244	Jehania	LN897454	P	Asia II-1	Cotton	MF599707	MF511898	-	-	-
245		OM281720	P	Asia II-1		-	MF511899	-	-	-
246		OM281721	P	Asia II-1		-	-	-	-	-
247		OM281722	P	Asia II-1		MF599627	MF511860	-	-	MF581618
248	Khairpur tamewali	LN835372	P	Asia II-1	Cotton	MF599621	MF511888	OQ747917	-	-
249		LN835406	P	Asia II-1		MF599622	MF511889	OQ747919	-	-
250		OM281723	P	Asia II-1		MF599623	MF511890	-	-	-
251		OM281724	P	Asia II-1		-	-	-	-	-
252	Yazman mandi	LN835402	P	Asia II-1	Cotton	MF599606	PZ018084	OQ747918	-	-
253		LN835403	P	Asia II-1		MF599607	PZ018085	-	-	-
254		OM281725	P	Asia II-1		MF599608	PZ018086	-	-	-
255		OM281726	P	Asia II-1		MF599609	-	-	-	-
256	Basti Ali Shah	LN832327	P	Asia II-1	Cotton	MF599689	MF511866	-	-	-
257		LN832328	P	Asia II-1		-	-	-	-	-
258	Lal sohanra	LN835404	P	Asia II-1	Chillie s	MF599610	PZ018087	OQ747920	-	-
259		LN835405	P	Asia II-1		MF599611	PZ018088	-	-	-
260		OM281727	P	Asia II-1		PZ044247	PZ018089	-	-	-
261		OM281728	P	Asia II-1		PZ044248	MF511841	-	-	-
262	Muslim town	LN835407	P	Asia II-1	Cotton	MF599624	MF511891	-	-	MF581621
263		LN897441	P	Asia II-1		MF599625	MF511892	-	-	-
264		LN897442	P	Asia II-1		OQ784583	PZ018090	-	-	-

265		OM281729	P	Asia II-1		PZ044249	PZ018091	-	-	-
266	Murad wala	LN832323	P	Asia II-1	Cotton	-	-	PZ012288	-	-
267		LN832324	P	Asia II-1		-	-	-	-	-
268	Panjnad	LN832329	P	Asia II-1	Cotton	MF599691	MF511830	-	-	-
269		LN832330	P	Asia II-1		MF599629	MF511831	-	-	-
270	Kot Sabzal	LN897440	P	Asia II-1	Cotton	MF599634	-	-	-	MF581583
271		OM281730	P	Asia II-1		MF599701	-	-	-	OQ747923
272	Vehari	HG315642	P	Asia II-1		PZ044250	PZ018092	-	-	-
273		HG764136	P	Asia II-1		PZ044251	PZ018092	-	-	-
274		HG764137	P	Asia II-1		PZ044252	PZ018092	-	-	-

Abbreviations used for endosymbionts are Sindh (S), Khyber Pakhtunkhawa (K), Punjab (P), *Arsenophonous* (A), *Cardinium* (C), *Hamiltonella* (H), *Rickettsia* (R), and *Wolbachia* (W).
